# Barriers to healthcare-worker adherence to infection prevention and control practices in British Columbia during the coronavirus disease 2019 (COVID-19) pandemic: A cross-sectional study

**DOI:** 10.1017/ice.2023.242

**Published:** 2024-04

**Authors:** Brooke T. Cheng, R. Ayesha Ali, Jun Chen Collet, Tara Donovan Towell, Guanghong Han, Dave Keen, Ka Wai Leung, Julie Mori, Jocelyn A. Srigley

**Affiliations:** 1 Faculty of Medicine, University of British Columbia, Vancouver, British Columbia, Canada; 2 Department of Mathematics and Statistics, University of Guelph, Guelph, Ontario, Canada; 3 Provincial Health Services Authority, Vancouver, British Columbia, Canada; 4 Fraser Health Authority, Surrey, British Columbia, Canada; 5 Interior Health, Kelowna, British Columbia, Canada; 6 Department of Pathology and Laboratory Medicine, BC Children’s Hospital and BC Women’s Hospital + Health Centre, Vancouver, British Columbia, Canada

## Abstract

**Objective:**

The coronavirus disease 2019 (COVID-19) pandemic highlighted the importance of robust infection prevention and control (IPAC) practices to maintain patient and staff safety. However, healthcare workers (HCWs) face many barriers that affect their ability to follow these practices. We identified barriers affecting HCW adherence to IPAC practices during the pandemic in British Columbia, Canada.

**Design:**

Cross-sectional web-based survey.

**Setting:**

Acute care, long-term care or assisted living, outpatient, mental health, prehospital care, and home care.

**Participants:**

Eligible respondents included direct-care providers and IPAC professionals working in these settings in all health authorities across British Columbia.

**Methods:**

We conducted a web-based survey from August to September 2021 to assess respondent knowledge and attitudes toward IPAC within the context of the COVID-19 pandemic. Respondents were asked to rate the extent to which various barriers affected their ability to follow IPAC practices throughout the pandemic and to make suggestions for improvement.

**Results:**

The final analysis included 2,488 responses; 36% of respondents worked in acute care. Overall, perceptions of IPAC practice among non-IPAC professionals were positive. The main self-perceived barriers to adherence included inadequate staffing to cover absences (58%), limited space in staff rooms (57%), multibed rooms (51%), and confusing messages about IPAC practices (51%). Common suggestions for improvement included receiving more support from IPAC leadership and clearer communication about required IPAC practices.

**Conclusions:**

Our findings highlight frontline HCW perspectives regarding priority areas of improvement for IPAC practices. They will inform policy and guideline development to prevent transmission of COVID-19 and future emerging infections.

The coronavirus disease 2019 (COVID-19) pandemic has required an intensive and rapidly changing infection prevention and control (IPAC) response in healthcare settings. Adherence to routine IPAC practices was known to be suboptimal prior to the pandemic, resulting in transmission of infections to patients and healthcare workers (HCWs).^1,2^ Potential barriers to adherence include HCWs being too busy, forgetting, having knowledge gaps, not believing that IPAC practices are effective, and lack of appropriate supplies or infrastructure.^3–5^

The importance of adherence to IPAC practices is even greater now that the healthcare system has been strained by the COVID-19 pandemic. Transmission of COVID-19 within healthcare settings has vast consequences, both at the individual level (infection-related morbidity and mortality) and system level (eg, closing areas where outbreaks are occurring, HCWs unable to work due to infections or exposures). This situation was exacerbated by community and household exposures of COVID-19 among HCWs, which caused even greater risk of transmission than the workplace.^6–8^ Therefore, it is imperative to understand the barriers to implementing IPAC measures to protect HCWs and patients from COVID-19 transmission.

We conducted a cross-sectional survey to assess the barriers to HCW adherence to IPAC practices in the province of British Columbia during the COVID-19 pandemic. We have previously published analyses from these data comparing acute care and long-term care and assisted living (LTC/AL) settings, showing that barriers were different and that strengthening IPAC programs in LTC/AL requires enhanced IPAC staffing and leadership, increased training and education, and improving access to personal protective equipment (PPE), hand hygiene, and cleaning products.^9^ Here we present a descriptive examination of the barriers to adherence to COVID-19 IPAC practices in various care settings among different types of HCWs in British Columbia.

## Methods

### Project setting and design

This project consisted of a cross-sectional, web-based, open survey administered in British Columbia. Eligible respondents included HCWs providing direct patient care who worked part- or full-time in acute care, LTC/AL, outpatient and community settings, prehospital care, and/or home care, as well as IPAC professionals who interacted with and educated those direct-care providers. For the purposes of this survey, direct patient care was defined as working in the patient environment (eg, entering patient rooms, face-to-face interaction with patients). To participate in the survey, respondents had to work at 1 of the 8 publicly funded health authorities in British Columbia as their primary work environment.

### Survey Development

The survey was based on existing IPAC survey tools and input from the multidisciplinary study team, made up of IPAC and workplace health professionals across multiple BC health authorities (Supplementary Material online). The survey included the following sections:

1. Knowledge assessment of COVID-19 IPAC practices
2. Potential barriers to COVID-19 IPAC practices, organized into 4 categories: perception, guidance and communication, infrastructure, and frontline work environment
3. Suggestions for how to overcome these barriers.

The knowledge assessment included selecting what PPE was indicated for patients with or under investigation for COVID-19 (with no aerosol-generating medical procedures). During the period referenced in the survey, provincial guidelines on the use of N95 respirators changed^10^ and certain health authorities implemented differing recommendations. Thus, selection of at least 1, medical mask or N95 respirator, was deemed correct.

A 5-point Likert scale was used by respondents to rank the level of importance of each barrier, and a free-text option was provided for additional barriers to be documented. Sections regarding knowledge assessment and perception of IPAC practices were only included for non-IPAC professionals, given that IPAC professionals would likely have greater knowledge of and more positive attitudes toward IPAC practices. The survey was pilot-tested by a convenience sample of non-IPAC and IPAC professionals from various health authorities and settings, who provided feedback on readability and content.

### Participant recruitment

The online survey was administered using the secure REDCap platform.^11^ Recruitment of participants occurred from August 11, 2021, to September 24, 2021, via staff newsletters and communication platforms of each health authority, as well as social media and public websites of affiliated organizations. To incentivize participation, 6 participants were randomly chosen to each receive a $50 Amazon gift card. Contact information of participants who wished to enter the drawing was not linked to survey responses.

Following promotion of the survey on health authority social media in August 2021, we received a high volume of suspicious entries over a very short period, suggestive of a survey bot attack. We temporarily closed the survey for 4 days and implemented additional measures to prevent fraudulent responses (Supplementary Materials online).

### Statistical analysis

Raw survey data were abstracted from the REDCap database. Data cleaning and recording was managed using Excel (Microsoft, Redmond, WA). Analyses were performed using R version 4.0.4 software (R Foundation for Statistical Computing, Vienna, Austria). For the ratings of barriers, affirmative responses (ie, moderately to greatly agree) were grouped together. Responses of “prefer not to answer” were excluded. We computed the proportion of respondents who agreed that each listed factor was a barrier, retrospectively stratified by job category and primary workplace. We excluded surveys that were incomplete, not fully submitted, or did not have a properly completed consent portion to exclude potential fraudulent responses. Furthermore, responses from IPAC professionals and other HCWs were combined, wherever possible, to minimize the impact of unsolicited entries on any healthcare group.

A list of prespecified responses and open-text responses were used to collect respondents’ suggestions to improve adherence to IPAC practices in the workplace. Thematic analysis of the qualitative survey responses was conducted independently by 2 members of the study team, adapted from the theoretical domains framework^12^ and based on the frequency of specific terms used in the open -text responses. Discrepancies between the 2 reviewers were adjudicated by a third team member. Where applicable, qualitative responses were reclassified to a prespecified recommendation or suggestion.

### Ethics approval

Based on the Provincial Health Services Authority project sorting tool,^13^ this project was determined to be a quality improvement intervention and involved minimal risk to participants. To address privacy concerns, no personally identifiable information was collected; the final survey and incentivization plan was reviewed by a privacy officer.^14^

## Results

### Demographics

Of 3,143 survey responses obtained through REDCap, 3,110 included consent to participate (participation rate, 99.0%) and 2,755 completed the survey with a timestamp (completion, 88.6%).^15^ Of those 2,755 completed surveys, 2,488 were from eligible participants and were included in the final analyses. It was not possible to determine the number of eligible HCWs; at the time of the survey, health authorities in British Columbia had >120,000 employees and >16,000 medical staff. Responses were received from every health authority in British Columbia. Participant demographics are shown in Table 1.

**Table 1.** Respondent Demographics Among 2,488 Eligible Respondents

Characteristic	No. (N = 2,488)	%
**Sex**		
Female	1,354	54.4
Male	1,035	41.5
Other	99	4.0
**Age group**		
<30 y	885	35.6
30–39 y	903	36.3
40–49 y	399	16.0
50–59 y	229	9.2
≥60 y	72	2.9
**Years worked in health care**		
≤5	902	36.3
6–10	816	32.8
11–15	343	13.8
≥16	427	17.2
**Job category**		
Nursing	750	30.1
Clinical support	684	27.5
Paramedic	95	3.8
Physician and other providers	63	2.5
Other	896	36.0
**Primary workplace**		
Acute care	896	36.0
Outpatient/Community	723	29.1
LTC/Assisted living	441	17.1
Home care	193	7.8
Mental health	128	5.1
Prehospital care	98	3.8
Other	9	0.4

Note: LTC, long-term care. Job categories included the following positions: nursing staff (nurses and care aides); clinical support (occupational therapy, physical therapy, social work, other allied health staff); physicians and other providers (nurse practitioners, midwives, and dentists); other staff (food services, housekeeping, and facilities operations).

### IPAC knowledge

Non-IPAC respondents were surveyed on their knowledge and practice of appropriate PPE and alcohol-based hand sanitizer use. Because respondents did not answer all questions, denominators for percentages varied (Table 2). Although 97% reported that they knew how to properly don and doff PPE, only 54% felt confident that they knew what PPE should be used for COVID-19 patients. Furthermore, 16% missed at least 1 required component (eg, gown, goggles, gloves, medical mask and/or N95 respirator) when asked to select what PPE was indicated for patients with or under investigation for COVID-19 (no aerosol-generating medical procedures). Furthermore, 18% incorrectly indicated that alcohol-based hand sanitizer does not effectively kill the SARS-CoV-2 virus.

**Table 2.** Percentage of Respondents Who Answered “Yes” to Questions on Knowledge of Proper IPAC Practices, Among the 1,130 Non-IPAC Respondents

Population	No. of Respondents (min, max)	Knows Correct PPE to Use	Knows How to Don/Doff PPE Properly	Alcohol-Based Sanitizer Effectively Kills SARS-CoV-2
Overall	(1,119, 1,129)	54	97	82
**Job category**				
Nursing	(592, 597)	63	99	81
Clinical support	(335, 339)	56	96	81
Paramedic	(42, 45)	2	96	91
Physician and other providers	(25, 26)	32	96	92
Other	(122, 123)	15	90	79
**Primary workplace**				
Acute Care	(658, 663)	60	98	81
Outpatient/ Community	(216, 219)	46	91	79
LTC/Assisted living	(107, 108)	56	97	86
Home care	(33, 34)	35	97	91
Mental health	(55, 56)	43	98	89
Prehospital care	(41, 42)	0	100	88
Other	8	25	100	88

Note: IPAC, infection prevention and control; LTC, long-term care; PPE, personal protective equipment.

To further understand how HCWs obtain IPAC knowledge, non-IPAC respondents were asked to select which sources of information they accessed throughout the pandemic. Information and guidance provided by one’s own health authority was the most commonly used resource (80%). Clinical support or nursing respondents consulted their health authority for information more than the other job categories. Among 461 respondents (41%) who reported conducting their own search for IPAC knowledge, most reported using the BC Centre for Disease Control website (86%), followed by materials provided by their own health authority (65%).

### Barriers to IPAC practices

#### Perception

Of non-IPAC respondents, overall perceptions of IPAC were generally positive (Table 3). A large majority believed that IPAC practices prevent transmission in the workplace (92%), are a priority compared to other work tasks (80%) and are their responsibility to implement (83%). Paramedics were least likely to perceive COVID-19 risk being low in the workplace (27%), compared to nurses (40%) and physicians/other providers (42%). However, paramedics, as well as physicians/other providers, reported approximately twice as often that other tasks had higher priority than IPAC practices, compared to clinical support and nursing staff (34% and 32% vs 12% and 17%, respectively). Home-care respondents had different perceptions than other workplace groups, being most likely to agree that workplace risk of COVID-19 was low (74%) and that IPAC practices were of lower priority compared to other tasks (53%) and were not their responsibility to implement (50%).

**Table 3.** Percentage of Respondents Who Moderately or Greatly Agreed that Each COVID-19 Perception Factor Affected Their Willingness to Follow IPAC Practices, Among the 1,130 Non-IPAC Respondents

Population	No. of Respondents (min, max)^a^	Risk of COVID-19 Is Low in Workplace	IPAC Practices Prevent Transmission of COVID-19 in Workplace	Other Tasks/Work Have Higher Priority Than IPAC Practices	Not My Responsibility to Ensure IPAC Practices for COVID-19 Are Implemented
Overall	(1,087, 1,123)	45	92	20	17
**Job category**					
Nursing	(582, 593)	40	92	17	10
Clinical support	(323, 338)	45	95	12	17
Paramedic	(43, 45)	27	89	34	23
Physician and other providers	(25, 26)	42	77	32	16
Other	(113, 122)	78	86	47	44
**Primary workplace**					
Acute Care	(639, 660)	41	93	15	11
Outpatient/Community	(208, 219)	57	89	18	18
LTC/Assisted living	(103, 107)	46	92	29	28
Home care	(34, 34)	74	91	53	50
Mental health	(54, 55)	46	96	35	24
Prehospital care	(39, 42)	31	85	34	26
Other	(8, 8)	50	88	13	25

Note: IPAC, infection prevention and control; LTC, long-term care.

a Respondents who experienced the barrier and provided a rating varied for each factor.

#### Guidance and communication

Among all 2,488 participants, the extent to which communication-related barriers affected ability to follow IPAC practices was mixed (Table 4). Nursing staff and acute-care respondents reported the highest percentages that frequent changes in IPAC guidance (62% and 56%, respectively) and confusing messages in the workplace (64% and 57%, respectively) moderately or greatly affected adherence to IPAC practices.

**Table 4.** Percentage of Respondents Who Moderately or Greatly Agreed That Each COVID-19 Guidance and Communication Factor Affected Their Ability to Follow IPAC Practices

Population	No. of Respondents (min, max)^a^	Frequent Changes in IPAC Guidance/Recommendations	Confusing Messages About IPAC Practices Within/From My Workplace	Contradictions in IPAC Guidance Between My Workplace and Other Sources
Overall	(1,672, 1,733)	51	53	50
**Job category**				
Nursing	(707, 731)	62	64	53
Clinical support	(643, 654)	46	49	46
Paramedic	(87, 89)	55	60	59
Physician and other providers	(57, 61)	49	47	53
Other	(839, 854)	45	45	49
**Primary workplace**				
Acute care	(834, 862)	56	57	48
Outpatient/Community	(677, 689)	47	50	49
LTC/Assisted living	(415, 426)	48	46	56
Home care	(182, 186)	47	54	55
Mental health	(119, 123)	54	51	42
Prehospital care	(92, 94)	46	67	40
Other	(8, 9)	63	44	33

Note: IPAC, infection prevention and control; LTC, long-term care.

a Respondents who experienced the barrier and provided a rating varied for each factor.

#### Infrastructure

Limited space was the most commonly experienced infrastructure-related barrier to following IPAC practices (Table 5). Nursing staff in particular frequently agreed that limited staff room capacity (71%) and multibed patient rooms (64%) were barriers. Overall, however, physicians and other providers and paramedics were most likely to agree that infrastructure-related barriers, including limited supplies and capacity issues, affected their IPAC practices compared to other job categories, whereas clinical support workers were least likely to be affected. Between 36% and 44% of respondents indicated that limited PPE, hand hygiene products, and/or cleaning products were significant barriers; this finding was primarily driven by nonacute-care respondents, with the highest rates in prehospital care, home care, and mental health.

**Table 5.** Percentage of Respondents Who Moderately/Greatly Agreed That Each Infrastructure Factor Affected Their Ability to Follow IPAC Practices

		Perceived Infrastructure Barrier, %									
Population	No. of Respondents (min, max)^a^	Limited PPE	Limited Hand Hygiene Products	Limited Cleaning Products	Limited Space Capacity in Staff Rooms	Prolonged Close Proximity to Patients	Multibed Rooms	Limited Space Capacity in Patient Dining Spaces	Cluttered Areas	Limited Dedicated Clean Space	Wandering Patients
Overall	(1,784, 2,369)	44	36	39	57	48	51	46	46	42	49
**Job category**											
Nursing	(398, 724)	45	35	37	71	58	64	55*	50	42	53
Clinical support	(465, 662)	36	30	33	54	36	42	40	37	35	41
Paramedic	(66, 90)	53	49	46	64	65	64	56	53	46	49
Physician and other providers	(44, 58)	46	42	46	66	63	60	50	60	61	54
Other	(805, 846)	48	39	45	46	44	45	45	46	46	53
**Primary workplace**											
Acute care	(462, 861)	34	29	30	66	51	56	45*	47	39	41
Outpatient/ Community	(552, 686)	46	35	43	51	42	46	43	43	42	49
LTC/Assisted living	(408, 422)	50	42	45	49	47	51	51	44	45	58
Home care	(171, 184)	51	44	46	48	48	47	42	49	45	52
Mental health	(101, 116)	57	41	47	53	44	45	49	48	43	60
Prehospital care	(71, 95)	63	47	48	61	67	54	61	51	52	57
Other	(2, 9)	29	14	25	67	33	33	50	50	50	50

Note: IPAC, infection prevention and control; LTC, long-term care.

a Respondents who experienced the barrier and provided a rating varied for each factor.

#### Frontline work environment

Regarding the work environment (Table 6), limited staff for covering absences was the most commonly cited barrier (58%) overall, particularly among nursing staff (79%), and those working in acute-care settings (70%). Feeling burnout or fatigue was common, especially among nursing staff (65%) and prehospital care (60%). For paramedics, many experienced difficulty functioning (66%) and fatigue from wearing PPE (63%). Notably, hand hygiene-related fatigue or concerns were among the least experienced barriers overall (37% and 44%, respectively).

**Table 6.** Percentage of Respondents who Moderately/Greatly Agreed that Each Frontline Work Environment Factor Affected Their Ability to Follow IPAC Practices

							Perceived Frontline Work Environment Barrier, %									
Population	No. of Respondents (min, max)^a^	Limited Time	Burnout/ Fatigue	Tired of Cleaning Hands	Concerns for Hand Skin	Tired of Wearing PPE	PPE Affects Function	Concerns for Skin	Ltd Cleaning Staff	Ltd Staff for Leave	Ltd IPAC Staff	Ltd IPAC Leadership	Ltd IPAC Communication	Ltd IPAC Training	Ltd Outbreak Knowledge	Ltd Patient Management Knowledge
Overall	(2229, 2398)	41	49	37	44	48	46	43	46	58	42	41	42	39	41	41
**Job category**																
Nursing	(676, 736)	41	65	34	48	56	52	49	55	79	47	44	44	38	43	42
Clinical support	(595, 667)	28	41	33	37	41	40	36	37	51	36	34	38	33	37	36
Paramedic	(81, 90)	48	53	50	49	63	66	46	59	67	54	51	47	39	44	38
Physician and other providers	(55, 60)	37	43	32	31	37	36	28	51	48	49	49	35	32	32	35
Other	(822, 863)	50	40	43	45	45	45	44	44	45	41	43	43	44	42	45
**Primary workplace**																
Acute care	(784, 878)	30	56	30	42	49	46	40	47	70	41	39	41	32	39	36
Outpatient/ Community	(653, 692)	45	41	40	44	45	45	43	43	47	42	39	43	40	42	41
LTC/Assisted living	(410, 427)	51	47	43	45	48	51	45	48	56	47	47	44	47	44	47
Home care	(177, 186)	48	41	46	41	47	45	47	49	51	39	47	45	42	45	47
Mental health	(110, 120)	39	47	48	43	45	35	45	44	46	38	34	32	37	29	38
Prehospital Care	(87, 97)	57	60	29	48	63	54	47	60	55	48	51	40	53	39	52
Other	(5, 9)	13	11	22	44	22	22	33	29	33	20	17	17	14	14	33

Note: IPAC, infection prevention and control; Ltd, limited; LTC, long-term care.

a Respondents who experienced the barrier and provided a rating varied for each factor.

### Suggestions for improvement

The 2 most popular suggestions for improvement (Table 7) were increasing IPAC leadership and support (60%) and addressing communication barriers (56%). A relatively high proportion of respondents also suggested improving availability of PPE and hand hygiene supplies (47%). Clarity of communication was a common suggestion, especially in the acute-care setting and among nurses and physicians/other providers.

**Table 7.** Suggestions Selected or Written by Respondents to Improve Ability to Follow IPAC Practices

		Suggested Improvements, %				
Population	No. of Respondents	Improved Access to PPE and Hand Hygiene	More IPAC Leadership and Support	More Frequent and Clear Communication	More In-Person Training and Education	Other Suggestions^a^
Overall	2,436	47	60	56	32	4
**Job category**						
Nursing	729	53	64	73	43	9
Clinical support	665	54	51	53	30	2
Paramedic	95	40	65	54	32	7
Physician and other providers	61	46	56	67	21	7
Other	886	37	65	44	26	1
**Primary workplace**						
Acute care	868	57	57	63	36	7
Outpatient/Community	712	39	64	54	30	2
LTC/Assisted living	433	44	61	46	31	2
Home care	191	44	64	47	23	1
Mental health	127	39	65	55	27	4
Prehospital care	98	48	52	68	45	7
Other	7	43	43	29	57	14

Note: IPAC, infection prevention and control; LTC, long-term care.

a Includes staffing and workload issues.

## Discussion

Overall, HCW perceptions of IPAC practices were positive; thus, targeted efforts to reinforce the importance of IPAC are not needed. Instead, providing an environment conducive to adherence to IPAC practices should be the central focus. Key barriers identified by respondents include inadequate staff to cover sick leave absences (58%), limited space capacity in staff rooms (57%), multibed rooms (51%), and confusing messages about IPAC practices (51%). In addition, barriers related to PPE (limited supply, PPE fatigue, and impaired function) were experienced by almost half of respondents, primarily in non–acute care settings. Common suggestions for improvement included improved IPAC leadership and support (60%), clear communication of IPAC guidelines (56%), and increased PPE and hand hygiene supplies (47%).

Many prepandemic studies examined facilitators and barriers to IPAC practices for respiratory infections, and they were summarized in a review.^4^ Similar to our findings, unclear and/or frequently changing IPAC guidelines, lack of training, and space limitations were commonly reported barriers. Several surveys of facilitators and barriers to IPAC practices among HCWs during the pandemic have been published, but most were limited in scope to only 1 or a few facilities and focused on self-reported compliance.^16–18^ A study in Alberta examined barriers to the use of PPE in long-term care settings,^5^ but to our knowledge, no studies in Canada have focused on HCWs from a variety of work environments. In a national survey conducted in Qatar, lack of PPE was the most commonly reported barrier.^19^ Although that was not the most common barrier in our survey, a similar proportion of respondents reported experiencing this scarcity. Similar to our results, perceptions of IPAC in Qatar were generally favorable. However, lack of knowledge and lack of training were far less commonly reported in Qatar compared to British Columbia, suggesting that there may be opportunities for improvement in our setting. Some studies conducted in lower-resource settings have similarly reported gaps in training, along with other barriers. For example, one study conducted throughout a province of Pakistan^20^ and another from a city in Ethiopia^21^ both found that overcrowding, lack of IPAC supplies such as PPE, and lack of training were major barriers. However, these latter surveys were all conducted earlier in the pandemic than ours, which may have affected the results. In addition, facilitators and barriers can vary by setting, so having local data is important to targeting quality improvement efforts.

Knowledge gaps were identified among respondents to this survey, with 16% missing at least 1 PPE component required for care of patients with COVID-19. Also, 18% were not aware that alcohol-based hand sanitizer is effective against COVID-19. In addition, almost half of respondents did not feel confident that they knew what PPE to use. Dissemination strategies, such as didactic education from IPAC staff, may have limited ability to improve adherence to IPAC guidelines.^22^ Peer auditing and staff coaching models as alternative behavior change methods may be effective ways to promote education and culture change surrounding IPAC practices.^23^ Such methods have been successfully implemented during the pandemic to improve PPE donning and doffing by HCWs.^24^

Confusing messaging about IPAC practices was a barrier experienced by approximately half of respondents and may have contributed to the identified knowledge gaps. IPAC teams in British Columbia should prioritize improved support for and communication to frontline staff. To better understand how to improve clarity of communication, further studies are needed to characterize which aspects of current practices are unclear. Development of communication frameworks to clarify target audiences and channels, as well as the use of novel strategies such as storytelling and social marketing, may be worth considering.^25,26^

Other commonly reported barriers related to physical infrastructure, PPE limitations, staffing, and sick-leave policies extend beyond the scope of IPAC teams but are important to address to optimize adherence to IPAC practices. For example, a higher proportion of private patient rooms was associated with a lower risk of healthcare-associated infections prior to the pandemic.^27,28^ During the pandemic, more crowded long-term care facilities were found to have more than double the rate of COVID-19 mortality.^29,30^ Addressing these operational challenges will require collaboration among multidisciplinary teams, including IPAC, across the healthcare system.

Notably, limited access to PPE was highlighted by a significant proportion of respondents (44%), and this was more commonly experienced in non–acute care settings. The survey asked respondents about experiences since the start of the pandemic, so it may be that limitations were experienced during the early waves. Shortages of PPE were common in 2020 for multiple reasons and were often more pronounced in non–acute care settings.^31,32^ In addition to limited supply, PPE fatigue and impaired function were commonly reported in our survey, consistent with published literature showing these to be barriers to IPAC practices.^33^

Further qualitative research to better understand barriers from the frontline worker experience would be an excellent complement to this study, particularly focused on groups that may have been underrepresented among respondents in our survey, such as physicians or care providers without regular access to computers (eg, environmental services staff). Our study also found differences in non–acute care work environments, such as greater limitations to PPE resources and lower perceived priority of IPAC practices. We have published our findings in LTC/AL settings,^9^ but further research to better characterize differences in other populations is warranted. In addition, cost-effectiveness studies would be helpful to quantify the impacts of improving hospital infrastructure on adherence to IPAC practices.

This study had several strengths, including being the first Canadian survey to comprehensively assess barriers to IPAC practices among HCWs during the COVID-19 pandemic with broad representation across job types and healthcare settings. However, this study had several limitations. First, the survey was closed between August 25 and 29, 2021, and eligibility for the cash prize was modified, in response to a bot attack. However, a sensitivity analysis did not find clear evidence of contamination (Supplementary Materials online). Second, the overall response rate was low. Overall, there are 120,000 employees of health authorities in British Columbia and >16,000 medical staff, although not all would have been eligible to participate (eg, administrative staff, other nonclinical workers). We did obtain responses from all health authorities and in proportions that were generally similar to their respective proportion of employees. Regardless, the sample of responses might not be fully representative of BC HCWs overall and results are further limited by selection bias given the online survey methodology. Furthermore, respondents had to be employees of a health authority in British Columbia so survey results would not apply to non–health authority settings, such as non–hospital-based outpatient clinics and privately owned and operated LTC/AL facilities. Additionally, our results are not likely generalizable to low- and middle-income countries. Finally, this survey was conducted >1 year into the COVID-19 pandemic, which may have helped to avoid bias from early supply and distribution issues of PPE and cleaning materials. However, responses may have also been affected by the widespread and ongoing promotion of IPAC. Regardless, there were likely challenges with barriers to following IPAC practices both during and prior to the pandemic. As such, our findings may be emphasizing a pre-existing issue that became more prevalent during pandemic times.

Our findings highlight frontline HCW perspectives on the priority areas needing improved IPAC interventions. As barriers were identified at the individual, institutional and systems levels of the healthcare system, we hope our findings will help fuel advocacy in multifaceted ways and stimulate changes. These results can inform future IPAC interventions for health authorities across the province, with the goal of preventing transmission of COVID-19 as well as future emerging infections.

## Supplementary material

To view supplementary material for this article, please visit https://doi.org/10.1017/ice.2023.242
